# Determinants of Delayed Presentation and Advanced-Stage Diagnosis of Breast Cancer in Africa: A Systematic Review and Meta-Analysis

**DOI:** 10.31557/APJCP.2021.22.4.1007

**Published:** 2021-04

**Authors:** Olayide Agodirin, Isiaka Aremu, Ganiyu Rahman, Samuel Olatoke, Julius Olaogun, Halimat Akande, Anya Romanoff

**Affiliations:** 1 *Department of Surgery, University of Ilorin and University of Ilorin Teaching Hospital, Ilorin, Kwara State. Nigeria. *; 2 *Department of Surgery, University of Ilorin Teaching Hospital, Ilorin, Kwara state. Nigeria.*; 3 *Department of Surgery, University of Cape Coast and Cape Coast Teaching Hospital, Cape Coast. Ghana. *; 4 *Department of Surgery, Ekiti State University, and Ekiti State University Teaching Hospital, Ado-Ekiti, Ekiti state. Nigeria.*; 5 *Department of Radiology, University of Ilorin and University of Ilorin Teaching Hospital, Ilorin, Kwara state. Nigeria. *; 6 *Department of Global Health and Health System Design, Icahn School of Medicine at Mount Sinai, New York, NY, USA. *; 7 *Global Cancer Disparities Initiatives, Department of Surgery, Memorial Sloan Kettering Cancer Center, New York, NY, USA. *

**Keywords:** Breast cancer, delay, advanced-stage Africa, meta-analysis

## Abstract

**Background/Objective:**

Breast cancer (BC) mortality is exceptionally high in Africa due to late presentation and advanced-stage diagnosis. Previous studies examining barriers to early BC presentation are markedly inconsistent, showing conflicting findings within and between African regions, making resource allocation and designing interventional campaigns challenging. Our objective was to assess the strength or magnitude of the association between determinants/risk factors and delayed presentation/advanced-stage diagnosis of BC in Africa.

**Methods:**

Electronic searches in PubMed, AJOL, Google, ResearchGate, ScienceDirect, and PubMed Central found eligible articles between 2000 and 2020. The meta-analytical procedure in Meta-XL used the quality effect model. I-squared (I^2^) above 75% indicated high heterogeneity. The summary effect size was the odds ratio with 95% confidence intervals.

**Results:**

The effect of socio-economic and demographic determinants on delay varies across African regions. Low level of education (1.63, 95% CI 1.01-2.63), and not performing breast self examination (BSE) (13.59, 95% CI 3.33-55.4) were significantly associated with delayed presentation. Younger patients had more significant delays in West Africa (WA, 1.41, 95%CI 1.08-1.85), and the reverse occurred in North Africa (0.68, 95%CI 0.48-0.97). Lack of BC knowledge (1.59, 95% CI 1.29-1.97), not performing BSE, or no history of undergoing clinical breast examination (CBE) (2.45, 95% CI 1.60-3.40), were associated with advanced-stage disease at diagnosis. Older patients had significantly more advanced disease in WA, and the reverse occurred in South Africa. Aggressive molecular BC subtypes [Triple negative (OR 1.62, 95% CI 1.27-2.06) or HER2 positive (1.56, 95% CI 1.10-2.23)] were significant determinants of advanced-stage diagnosis.

**Conclusion:**

Promoting early presentation and reducing advanced-stage BC throughout Africa should focus on modifiable factors, including providing quality education, improving breast health awareness and BC knowledge, and developing strategies to increase BSE and CBE. Interventions targeting socio-demographic determinants should be context-specific.

## Introduction

Cancer of the breast is a disease of public health importance, accounting for more than 2 million new cases, 11.6% of all new cancers, and over 600,000 deaths, 6.6% of all cancer-related mortalities in 2018 (Bray et al., 2018). There is a disproportionate mortality to incidence ratio of breast cancer (BC) in Africa (Bray et al., 2018) due to long delays with presentation intervals longer than six months in most reports (Espina et al., 2017) and over 70% of patients diagnosed with advanced-stage disease (Jedy-Agba et al., 2016b). Consequently, raising the propensity for early help-seeking and lowering the probability of late-stage diagnosis is critical to improving BC outcomes in Africa. 

Studies examining barriers to early presentation and early diagnosis of BC in Africa show conflicting associations within and between regions of Africa (Mousaet al., 2011; Ibrahim and Oludara, 2012; Ayoade et al., 2015; Benbakhtaet al., 2015; Pace et al., 2015; Akinkuolie et al., 2016), thus making resource allocation and designing of interventional campaigns challenging. Furthermore, a study of breast cancer-specific survival in the US found that socio-economic determinants were specific to the white race (Agarwal et al., 2017), noting that interventions based on economic status might not influence outcomes among black women. Another study reported that the association between demographics and poor outcomes in black women might be related to tumor biology (Iqbal et al., 2015). 

Identifying the most influential risk factors for BC delays and advanced-stage disease presentation will help in designing more effective interventions. Therefore, this meta-analysis aimed to assess the strength of association between the determinants/risk factors and delayed presentation of BC or advanced-stage diagnosis in Africa by comparing the prevalence of the determinants between late and early presenters. Our ultimate goal is to provide data to understand each determinant’s relevance, thus aiding the planning of future campaigns against BC in Africa.

## Materials and Methods

This research was conducted according to the MOOSE (meta-analysis of observational studies in epidemiology) guidelines. The need assessment and preliminary literature review [in PubMed, Cochrane library, and Prospero, reference ID CRD42020150932], confirmed there was no similar meta-analysis conducted previously or ongoing. The full literature search was conducted in PubMed.gov from August 19, 2020, to September 3, 2020, using the search terms “delay OR late AND stage AND presentation AND breast cancer Africa.” Hand-search was done on African Journal Online (AJOL), Google, Google Scholar, ScienceDirect, PubMed central, ResearchGate, and Academia. The Snow-balling search was performed in the reference list of original articles and already published systematic reviews. We sent exclusive electronic request messages to authors for full articles not available online or to clarify data. 

*Article selection and data extraction*

Author AO screened articles eligible for full review using the title and abstract. Authors AO and AI independently conducted the full-article review and data extraction using predefined Population, Intervention, Control, Outcome, Time, Study design (PICOTS) criteria shown in Table 1 in the appendix and supplementary file. The authors discussed to resolve any disagreement.

*Quality assessment*

We adapted the quality assessment variables in the STROBE checklist to define the quality assessment criteria, placing a premium on variables that showed face and content validity of a study primarily designed to describe determinants of late presentation of breast cancer (quality assessment table in Supplementary file). The maximum score was ten, and the minimum was zero. 

*Statistical analysis*

The primary outcome was the odds ratio (OR) of the risk factors in delayed presentation or advanced-stage versus the early-stage presenters. The summary odds ratio (OR) was calculated in the meta-analytical procedure, with a 95% confidence interval for all determinants or risk factors where a minimum of two observations were available, and a simple OR with a 95% confidence interval when only one observation was available. The analysis was reported as a continent-wide result when at least two regions were represented. The meta-analytical procedure was in MetaXL add-in for Microsoft Excel http://www.epigear.com with the quality effect model, using the quality score derived for each study, thus assigning higher weights to studies with better quality scores. I-squared (I2) above 50% indicated moderate heterogeneity and above 75% high heterogeneity. Subgroup analyses and sensitivity analyses were conducted to explain significant heterogeneity. 

## Results

**Figure 1:**
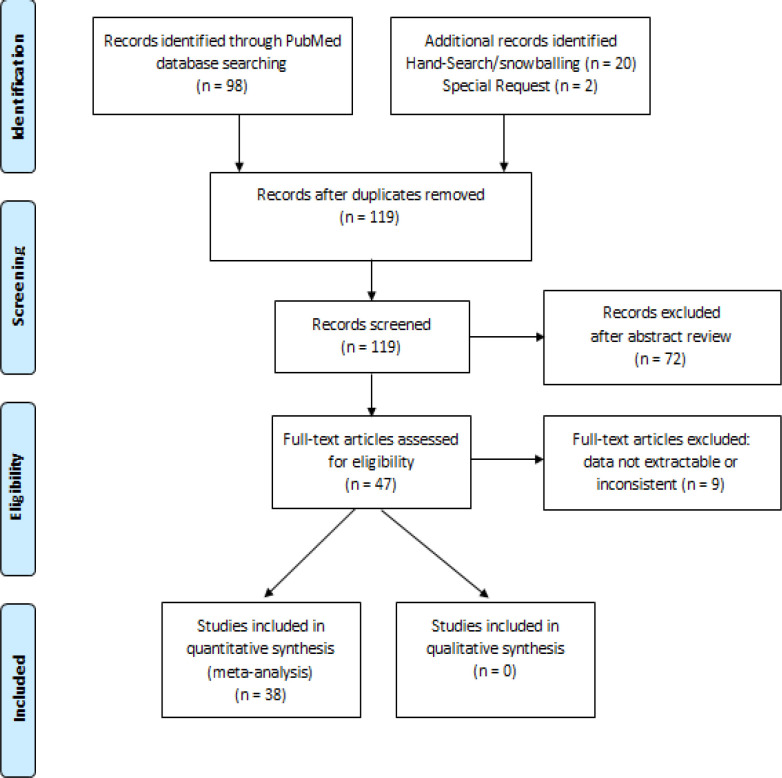
Flow Chart Diagram of Article Screening

The search in PubMed found 97 articles. Eighty-one were excluded after the title, abstract, and full-text review (Figure 1). Special requests sent to 4 authors yielded two additional articles (Benbakhtaet al., 2015; Rayne et al., 2019) while the other two could not be reached (Mody et al., 2013; Youngblood et al., 2020). Hand-searching and snow-balling yielded 21 more articles. 

In total, 38 articles contributed to the quantitative meta-analysis. In twelve articles, 16,347 subjects contributed to the analysis of the determinant of delayed presentation, and in 31 articles, 45,177 subjects contributed to the analysis of advanced-stage determinants. One article was published in French (Benbakhta et al., 2015); all others were English. Sixteen countries across all regions of Africa [Central Africa- CA, East Africa- EA, North Africa-NA, Southern Africa- SNA, West Africa- WA] contributed data; 12 countries contributed data in a single-center study while two contributed data as part of multination studies (McKenzie et al., 2018; Islami et al., 2015). 

Nine of 38 scored seven and above in the quality assessment, while 21 scored five and above (Quality Table, Supplementary file). The rationale in 22 (58%) of included articles was to describe the determinant of delayed presentation or advanced-stage diagnosis. Other articles found during our extensive snow-balling and hand-search were descriptive studies of presentation patterns or tumor biology reporting on determinants of delay or advanced-stage diagnosis. The articles reporting on delay described it as the interval between symptom recognition and first visit to the study center except two that defined delay as the interval between symptom recognition and diagnosis at the study center (Ermiah et al., 2012; Grosse et al., 2018).

*Determinants of delayed presentation*

Education level was the only socioeconomic variable that influenced delay in the overall analysis; women with lower education had longer delays to presentation (OR 1.63, 95% CI 1.01-2.63) (Table 3). In the age influence analysis, six studies used 40 years as a cut-off, two used 50, one used 46, and two used 45 years. Sensitivity analysis, including only studies with 40 years cut-off, also did not show a significant association (OR 0.96, 95% CI 0.67-1.67). In the regional analysis, younger patients delayed longer in WA (OR 1.38, 95% CI 1.02-1.86), while the reverse occurred in NA (OR 0.68, 95% CI 0.48-0.97) (Table 3).

Women who did not perform breast self-examination (BSE) had 13 times higher odds of delayed presentation (Table 3). BC awareness (OR 1.17, 95% CI 0.52-2.63), family history of BC (OR 2.88, 95% CI 0.50-16.53), absence of a breast mass (OR 2.68, 95% CI 0.26-28.9) or first visiting alternative healthcare practitioners (OR 2.36, 95% CI 0.4-12.18) did not significantly influence delay. A single study analysis for risk of delay among patients with low BC knowledge (Ayoade et al., 2015) (OR 1.61, 95% CI 1.20-2.17) showed a significant association with delay. Other single studies showed that misinterpreting breast cancer symptoms as breast infection (Grosse et al., 2018) (OR 6.11, 95% CI 1.49-25.1) and having a smaller mass (Agodirin et al., 2020), (OR 1.56, 95% CI 1.0-2.43) were associated with delay. 

*Determinants of advanced-stage diagnosis*

Socio-economic and demographic indicators did not significantly affect advanced-stage diagnosis in the overall analysis. The influence of age, level of education, and marital status varied across the regions. WA’s younger patients had reduced risk, while SA’s younger patients had an increased risk of advanced-stage diagnosis. Unmarried women and women with lower education levels had the more advanced disease in WA (Table 4). In the single study analysis, religion (Muslim vs. Christian) (Jedy-Agba et al., 2017) (OR 1.10, 95% CI 0.54-2.28), or socioeconomic status (low vs. high) (OR 1.22, 95% CI 0.76-1.94), did not significantly affect advanced-stage diagnosis (Table 4). 

Women who did not perform BSE, those who never experienced clinical breast examination (CBE), and those who lacked BC knowledge had more advanced disease. First visiting an alternative healthcare practitioner and visiting multiple medical personnel before arriving at the oncology center did not significantly affect BC stage. Delay of three months between symptom detection and diagnosis center was significant in NA, but not in the overall analysis. In a single study analysis (Joffe et al., 2018), visiting several healthcare providers before arriving at the oncology center did not significantly affect the disease stage (>1HCP vs. 1 HCP (OR 1.33, 95% CI 0.92-1.93)). Family history of BC and absence of a breast mass in the initial symptomatology did not significantly influence the disease stage. 

**Figure 2:**
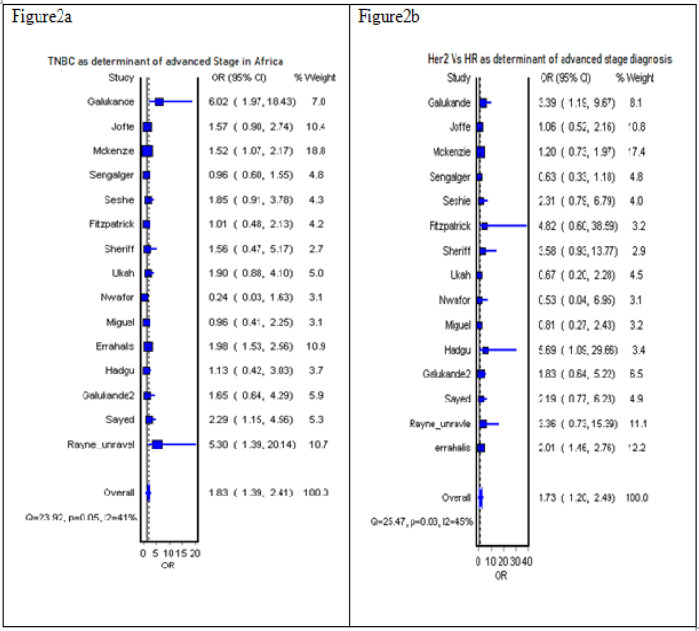
Forest Plot Showing the Analysis of Molecular Type of Tumor and Risk of Advanced-Stage Diagnosis

Considering non-modifiable factors, triple-negative (OR 1.83, 95% CI 1.39-2.41) or HER2 positive BC (OR 1.73, CI 1.20-2.49) was associated with more advanced stage at diagnosis (Figure 2), whereas tumor grade did not significantly affect the disease stage. In a single study analysis (Rayne et al., 2019), there was no significant association (OR 1.69, 95% CI 0.88-3.27) between black race and advanced-stage diagnosis. 

**Table 1:** PICOTS Article Screening Criteria

Participants/Population	Freely available articles reporting on the risk factors linked to advanced-stage presentation or delayed presentation among female breast cancer (BC) patients in Africa. Studies involving more than 5% of male patients were excluded.
Intervention	Not applicable
Control/comparison	The odds ratio of the determinants or risk factors for Advanced-stage diagnosis or delayed presentation in late presenters (Case) compared to early presenters (Control). Early-stage diagnosis was defined as stage 0, I, or II or diagnosis with primary tumor ≤5cm and Advanced-stage diagnosis as stage III or IV disease or with primary tumor >5cm diameter. Based on the predominant pattern of reporting in African literature, early help-seeking was defined as presentation to the study center within three months of symptom recognition, and late help-seeking was defined as presentation after three months of symptom recognition. The absolute counts of risk factor(s) in the late presenters (case) and early presenters (control) had to be published or extractable for a study to be eligible.
Outcomes	Socio-demographic risk factors: Age, marital status, educational status, distance to help care provider Knowledge, awareness, and family history of BC. Pattern of help-seeking: Visiting alternative care first, number of health care providers visited. Breast health behavior: Performing Breast Self-Examination Biologic risk factors: Tumor grade and immunohistochemistry receptor status. The definition /analysis of the determinants: Association with age was analyzed as younger population or older population as defined by the study hence any cut off between 40 and 50 years was acceptable as the emphasis was on the pattern of presentation of the younger compared to the older population age group in the study rather than the exact age defined as young or old. Older age was the reference level Marital status was analyzed as married or unmarried (comprising single, widows, separated or divorced). Married was the reference level. Educational status was analyzed as no education / primary education level versus secondary/tertiary level education. Secondary/tertiary level was the reference level Being unemployed was compared to being employed, being employed was the reference level. Socio-economic class was analyzed as low vs intermediate / high. Rural dwelling, living remote to study center (>10km away or >1hr drive ) was compared to urban dwelling, living close to study center <10km away or <1hr drive) (reference level). Consulting alternative medicine first was compared to consulting orthodox health care provider first(reference level). Seeing more than one Health care provider was compared to seeing only one healthcare provider(reference level). Triple negative status was compared to Hormone receptor positive(luminal A and B) status (reference level) and HER2 positive status was compared to Hormone receptor status (reference level). Classification of determinants The determinants of advanced-stage presentation or delayed presentation were classified as demographic; age, social; including the marital status, educational status, economic indicator; including employment status, and level of income. Awareness and knowledge, breast health behavior and screening practices, the pattern of health-seeking, and the tumor biology comprising tumor grade and molecular receptor status.
Time	Articles published between January 2010 and August 2020. Articles including data earlier than 2000 were excluded
Study design	We included observational studies (prospective, retrospective, and surveys) reporting on risk factors for late-stage diagnosis or delayed presentation of BC in African. Qualitative studies and studies including more than 5% male population were excluded. The language was not an exclusion criterion

**Table 2:** Article Characteristics Summarizing the Year, Country, Region, Institution, Design, and Study Level Summary Statistics of Eligible Articles

First Author	Year	Country	United Nations Region	Race	Period	Location of hospital	Design	N	Study level age statistic. range/mean/median
Adly et al(Adly, Hewedi and Mokhtar, 2010)	2010	Egypt	NA	NS	2007-2009	University for Science & Technology Hospital & Ain Shams University Hospital			
Agbo(Agbo, Khalid and Oboirien) et al	2014	Nigeria	WA	NS	2007-2011	Usmanu Dan Fodio Teaching Hospital Sokoto	retro	816	NS/48.2/NS
Agodirin(Agodirin, Olatoke, Rahman et al., 2017) et al	2018	Nigeria	WA	NS	2016-2018	Multicenter	survey	100	26-80/50.5/NS
Akanbi(Akanbi, Oguntola, Adeoti et al., 2015) et al	2015	Nigeria	WA	NS	2012-2014	NS	cross-	120	NS
Ayoade(Ayoade, Salami, Agbola et al., 2015) et al	2015	Nigeria	WA	NS	2011-2014	Olabisi Onabanjo University Teaching Hospital Sagamu	survey	113	NS/47.8/NS
Benbakhta(Benbakhta, Tazi, Benjaafar, Khattabi and Maaroufi, 2015)	2013	Morocco	NA	NS	2012-2013	INO Rabat	cross	200	25-82/49.1/
Brinton(Brinton, Figueroa, Adjei et al., 2017) et al	2017	Ghana	WA	NS	NS	Korle Bu Teaching Hospital & Komfo Anokye Kumasi & Peace and Love Kumasi	case	1184	18-74/NS/NS
Burson(Burson, Soliman, Ngoma et al., 2010) et al	2010	Tanzania	EA	NS	2007-2009	Ocean Road Cancer Institute Dar es Salam	retro	488	NS/43.4/NS
Cacala(Cacala and Gilart, 2017)	2017	South Africa	SNA	NS	2014	Grey's Hospital KwaZulu Natal Pietermaritzburg	pros	172	23-100/56/NS
Elima-jedy(Jedy-Agba, McCormack, Adebamowo and Dos-Santos-Silva, 2016a) et al	2017	Nigeria	WA	NS	2014-2016	Multicenter	pros	316	24-86/45.4/NS
Ermiah(Ermiah, Abdalla, Buhmeida et al., 2012) et al	2012	Libya	NA	NS	2008-2009	National Oncology Institute Sabratha	survey	200	22-75/45.4/NS
Errahhali(Elidrissi Errahhali, Elidrissi Errahhali, Ouarzane et al., 2017) et al	2017	Morocco	NA	NS	2005-2012	Hassan II regional oncology center	retro	2406	NS/48.7/NS
Fitzpatrick(Fitzpatrick, Rendi, Kiviat et al., 2019) et al	2018	Senegal	WA	NS	2001-2016	Dantec Hospital	retro	197	NS/47/NS
Gabremariam(Gebremariam, Addissie, Worku et al., 2019) et al	2019	Ethiopia	EA	NS	2017-2018	Multicenter	cross	441	NS/44.4/NS
Galukande(Galukande, Mirembe and Wabinga, 2014) et a	2014	Uganda	EA	NS	2008-2011	Mulago Hospital Kampala	retro	201	22-87/46.5/45
Galukande(Galukande, Wabinga and Mirembe, 2015) et al2	2015	Uganda	EA	NS	2004-2012	National Institute Oncology Sabratha		200	22-75/NS/NS
Gross-frie(Grosse Frie, Kamate, Traore et al., 2018) et al	2018	Mali	WA	NS	2016	University Hospital Bamako	survey	64	NS/45/NS
Gueye et al(Gueye, Gueye, Diallo et al., 2017)	2017	Senegal	WA	NS	2014	Aristide Le Dantec Teaching Hospital Dakar	obser	259	NS/NS/NS
Hadgu(Hadgu, Seifu, Tigneh et al., 2018)	2018	Ethiopia	WA	NS	2012-2015	Oncology Center Tikur Ambessa Specialized Hospital & Paul's Hospital Millenium Medical College	retro	114	25-75/43/40
Islami(Islami, Lortet-Tieulent, Okello et al., 2015a)	2015	Congo & Ivory Coast	WA	NS	2008-2009	Cancer Registry Abidjan Ivory Coast & Brazzaviille Congo	retro	280	NS/NS/NS
Joffe(Joffe, Ayeni, Norris et al., 2018) et al	2018	South Africa	SNA	NS	2015-2016	Chris Hani Baragwanath Academic Hospital Hospital Soweto	survey	499	NS
Kholer(Kohler, Moses, Krysiak, Liomba and Gopal, 2015) et al	2015	Malawi	EA	NS	2011-2013	Kamuzu Central Hospital Lilongwe	retro	198	12-89/NS/34
Marcus(Marcus, Lunda and L, 2013)	2013	South Africa	SA	NS	2007-2010	Sebokeng Hospital, Sedibeng Municipal district , South Gauteng	retro	103	34-83/59/NS
McKenzie(McKenzie, Zietsman, Galukande et al., 2018)	2017	multicenter	SSA	blk/nblk	2014-2017	Multicenter	cross	1795	NS/NS/NS
Miguel(Miguel, Lopes, Ferreira et al., 2017) Africa	2017	Angola	CA	NS	2011-2014	Angola Institute of Cancer Control Luanda & clinica Sagrade esperanca	pros	140	24-84/47/NS
First author	Year	Country	United Nations Region	Race	Period	Location of hospital	Design	N	Study level age statistic. range/mean/median
Mousa(Mousa, Seifeldin, Hablas, Elbana and Soliman, 2011) Africa	2011	Egypt	NA	NS	2009-2010	Tanta Cancer Center Gharbiah Province	survey	163	NS
Muchuweti(Muchuweti, Nyandoro, Muguti and Muchaziwepi, 2017) et al	2017	Zimbabwe	EA	NS	2010-2013	Parirenyatwa Group of Hospital Harare	pros	73	NS
Nwafor(Nwafor and Keshinro, 2015) et al	2012	Nigeria	WA	NS	2009-2013	MeCure Health Limited Lagos	retro	48	29-78/49.5/NS
Odongo(Odongo, Makumbi, Kalungi and Galukande, 2015)	2015	Uganda	EA	NS	2014	Mulago National referral Hospital	cross	162	NS/45.1/44
Olanrewaju(Olarewaju, Oyekunle and Bamiro, 2019)	2019	Nigeria	WA	NS	2018	University College Hospital Ibadan	cross	275	NS/49/NS
Rayne et al (Rayne, Schnippel, Firnhaber et al., 2017)	2017	South Africa	SNA	blk (85) nblk (170)	2011-2013	Johannesburg	survey	263	18-86/NS/52
Rayne et al2 (Rayne, Schnippel, Grover et al., 2019)	2018	South Africa	SNA	NS	2016-2017	University of Witwatersrand & National Health Laboratory system	survey	231	NS/NS/56
Sayed et al(Sayed, Moloo, Wasike et al., 2018)	2018	Kenya	EA	NS	2012-2015	Multicenter	survey	846	NS/48/NS
Sengal(Sengal, Haj-Mukhtar, Elhaj et al., 2017) et al	2017	Eritrea	EA	NS	2011-2015	University of Gezira	retro	562	NS/NS/NS
Seshie et al.(Seshie, Adu-Aryee, Dedey, Calys-Tagoe and Clegg-Lamptey, 2015)	2015	Ghana	WA	NS	2011-2012	Korle Bu Teaching Hospital		165	24-77/52.2/NS
Stapleton (Stapleton, Mullan, Dey et al., 2011) et al	2011	Egypt	NA	NS	2007-2008	National Cancer Institute of Cairo University & Tanta Cancer Center Nile Delta	cross	343	NS/NS/NS
Tazzite(Tazzite, Jouhadi, Saiss, Benider and Nadifi, 2013) et al	2013	Morocco	NA	NS	2009	Oncology Centre, Ibn Rochd University Hospital Casablanca	retro	570	NS/47.07/NS
Ukah et al(Ukah, Chianakwana, Ebubedike et al., 2017)	2017	Nigeria	WA	NS	2010-2015	Nnamdi Azikwe University Teaching Hospital Nnewi	pros	397	NS/46.3//NS

NS, Not Specified; Regions, CA, Central Africa; EA, East Africa; NA, North Africa; WA,West Africa; SNA, Southern Africa; Race, blk-Black; nblk-Nonblack; Design, Case- Case Control, Cross- Cross-sectional, Pros-Prospective, Retro-Retrospective, Survey, Questionnaire based Survey;

N, number of subjects

**Table 3:** Summary Estimates of the Odds Ratio of Determinants of Delayed Presentation: Case represents subjects presenting in advanced stages (Stages III and IV), and control represents subjects presenting in early stages (Stages 0, I and II).

Variable (no of articles )	Case (n)	Control (n)	Pooled OR	95%CI	I-Squared	P-value
Demographic and social						
Age, overall (10)	Young (764)	Old (1158)	1.04	0.80-1.41	42%	0.08
Age, sensitivity (6)	<40 (477)	>40 (756)	1.07	0.71-1.63	42	0.12
Age, WA (4)	Young (406)	Old (267)	1.41	1.08-1.85	0%	0.67
Age, NA (3)	Young (270)	Old (141)	0.68	0.48-97	0%	0.59
Age, SA (2)	Young (88)	Old (168)	0.92	0.35-2.39	14	0.28
Awareness, overall (3)	Unaware (194)	Aware (276)	1.17	0.52-2.63	72%	0.03
Awareness, WA(2)	Unaware (136)	Aware (252)	1.01	0.25-4.07	82%	0.02
Family history, overall (2)	Negative (373)	Positive (34)	2.88	0.50-16.53	77%	0.04
Alternative healer, WA (2)	Alternative first (238)	Hospital first (82)	2.36	0.40-12.18	49%	0.16
BSE overall (2)	Not perform (242)	Perform (22)	13.59	3.33-55.4	0%	0.6
Symptomatology, overall (2)	No lump (90)	Lump (268)	2.68	0.26-28	94	0
Education, overall (8)	Low (696)	High (931)	1.63	1.01 -2.63	63%	0.01
Education, WA (4)	338	131	1.26	0.76-2.08	59%	0.06
Education, SA (2	133	120	2.39	1.08-5.29	0	0.8
Education, NA (2)	225	137	2.98	0.98-9.08	82%	0.02
Economic indicators						
Employment status, overall (5)	Unemployed (380)	Employed (389)	1.2	0.5-2.85	74%	0.01
Employment, WA (2)	Unemployed (158)	Employed (181)	0.91	0.2-4.13	83%	0.01
Employment, SA (2)	111	15	1.61	0.06-32.5	84%	0.01
Income, WA (3)	Low (385)	High (272)	1.38	0.94-2.04	0%	0.53
Family history, overall (2)	Negative (373)	Positive (34)	2.88	0.50-16.53	77%	0.04
Marital status, overall (8)	Unmarried (492)	Married (1003)	0.93	0.60-1.43	58%	0.02
Marital status, WA (4)	Unmarried (197)	Married (521)	0.96	0.41-2.27	79%	0.01
Marital status, NA (2)	105	48	0.92	0.58-1.44	0%	0.79
Marital status, SA (2)	160	28	0.74	0.27-2.07	45	0.18
Location overall (6)	Rural (473)	Urban (503)	1.59	0.82-3.07	76%	0
Location, WA (3)	Rural (189)	Urban (246)	1.51	0.91-2.94	12%	0.32
Location, NA (2)	Rural (150)	Urban (205)	2.09	0.28-15.8	91%	0

BSE, Breast Self Examination; NA, Northern Africa; SA, South Africa; WA, West Africa

**Table 4:** Summary Estimates of the Odds Ratio of Determinants of Advanced-Stage Presentation: Case represents subjects presenting in advanced stages (Stages III and IV), and control represents subjects presenting in early stages (Stages 0, I and II). Single Study analyses are identified as first author et al

Variable (no of articles)	Case (n)	Control (n)	Pooled OR	95%CI	I-Squared	P-value
Demographic and social						
Age, overall (9)	Young(1307)	Old (1470)	0.54	0.23-1.23	90%	0.01
Age, WA (4)	785	644	0.3	(0.12-0.73)	72%	0
Age, SA (2)	129	594	1.91	1.05-2.73	72	0
Age, EA (2)	194	133	0.2	0.01-4.36	92	0
Age, Benbakhta et al	91	103	0.54	0.31-0.95		
Education, overall (7)	Low (1645)	High (1929 )	1.26	0.89-1.79	76%	0.01
Education, WA (2)	161	56	2.02	1.37-2.99	0%	0.71
Education, SA (2)	267	683	0.9	0.56-1.44	48	0.16
Marital status, overall (6)	2115	2293	0.97	0.59-1.60	87%	0.01
Marital status, WA (2)	510	581	1.62	1.17-2.25	0%	0.76
Marital status, (SA)	529	431	1.06	0.8-1.41	0%	0.6
Burson et al	96	167	1.21	0.49-3.01		
Location, overall (7)	Rural (1605)	Urban (2427)	1.41	0.98-2.02	58%	0.01
Location, WA (4)	322	1238	1.11	0.86-1.64	0%	0.53
Location, EA (2)	153	76	2.9	0.49-17.16	76%	0.04
Location, SA (2)	278	171	2.01	0.97-4.17	69%	0.07
Economic indicator						
Employment status, overall (2)	Unemployed (230)	Employed (282)	1.02	0.41-2.57	55%	0.14
Engagement in screening practices						
BSE/CBE, overall (3)	755	254	2.45	1.6-3.40	0%	0.53
BSE, WA(2)	199	142	2.03	1.20-3.26	0%	76
Pattern of Help-seeking						
Alternative first, WA	35	336	1.44	0.41-5.14		
Symptomatology, overall (2)	No lump (49)	Lump (579)	1.12	0.29-4.26	63	0.1
Number of HCP	>1 HCP	1HCP	1.02	0.41-2.57	55%	0.14
Overall(2)	238	284				
Delay >3months, overall (8)	2276	1041	1.89	0.91-3.95	86%	0
Delay, NA (4)	343	468	2.25	1.60-3.16	0%	0.67
Awareness/Knowledge						
Family history, overall (3)	Negative (838)	Positive (156)	1.2	0.64-3.33	63%	0.07
Awareness, overall (2)	384	1690	2.21	1.68-2.92	0%	0.42
Knowledge of BC, overall (2)	735	1950	1.59	1.29-1.97	8%	0.3
Biology						
Grade overall (3)	high (332)	Medium/low (607)	1.25	0.91-1.71	0%	0.62
HER2 status, overall (3)	HER2 positive (370)	Hormone Positive (3897)	1.73	1.20-2.45	45	0.03
TNBC, overall (3)	TNBC (1149)	Hormone positive (3897)	1.83	1.39-2.41	41%	0.05

BC, Breast Cancer; BSE, Breast Self Examination; CBE, Clinical Breast Examination; HCP, Health Care Provider; NA, Northern Africa; SA, South Africa; WA, West Africa

## Discussion

Downstaging breast cancer is a high priority in Africa, and understanding the determinants of delayed presentation and advanced-stage diagnosis is critical to this endeavor. Our previous meta-analysis ordered the prevalence of perceived risk factors for delayed presentation, from already identified themes, among late presenters only. The current meta-analysis aggregated Africa’s data to compare the socio-economic, demographic, and biologic determinants of delayed presentation or advanced-stage diagnosis between early and late presenters and adds to the existing literature by describing each determinant’s significance. In the overall analysis, low education and not performing BSE increased the risk of delayed presentation significantly. Lack of BC knowledge, not performing BSE/ no history of undergoing Clinical Breast Examination and TNBC or HER2 positive BC, significantly increased the risk of advanced-stage diagnosis. 

Similar to prior research (Montella et al., 2001), we found an increased risk of delay among younger women in WA. Paradoxically, there was a reduced risk of advanced-stage diagnosis in the same population. The reason for this paradoxical trend is unclear. It might be due to planned behavior underpinned by sociocultural issues, such as fear of diagnosis and treatment, lack of trust in hospital, and unfavorable expectations of treatment outcome (Bish et al., 2005). While older persons might engage in screening and present early due to the perception of a higher risk of cancer (Deeks et al., 2009), they might also delay when they underestimate their symptoms (Innos et al., 2013) or when they show altruistic behavior, keeping the information from their relations to prevent them from worrying (Zhang et al., 2019). In contrast, younger persons might delay diagnosis and treatment due to unwanted changes in their bodies.

These regional differences suggest that age-directed messaging might be necessary for different regions. Future research should explore selective messaging that might influence patient subpopulations, such as the cluster of deliberate delayers already identified in Nigeria (Ayoade, et al., 2015; Agodirin et al., 2019). Reports show that tailored, individual messaging has potential to improve cancer awareness more than general information (Austoker et al., 2009; Linsell et al., 2009; Campbell et al., 2016), and individual messaging enhances BC knowledge and performance of BSE (Linsel et al., 2009; Campbell et al., 2016). 

This study showed a strong association between a low level of education and advanced-stage diagnosis. Similarly, in a population-based study in India, Mathew et al. found a strong association between education and early breast and cervical cancer diagnosis (Mathew et al., 2019). In explaining the association, Jedy-Agba et al., (2017) in Nigeria linked higher education levels to BC knowledge and believing in curability. Additionally, higher education levels might increase the likelihood of comprehending the health campaign messages with terminology that is not representative of local dialects. 

The level of education in Africa is transitioning with increasing school attendance, but the quality is lacking. Less than 7 percent of late primary school pupils in Sub-Saharan Africa achieve the appropriate reading proficiency and the majority are unable to comprehend simple written sentences (Sow, 2017). This may result in decreased comprehension of cancer campaign messaging. Females and children in low-income families are particularly disadvantaged (Ali et al., 2008; Kazeem et al., 2010; The Africa-American Institute, 2015), and much can be done by increasing the public spending on education and providing quality education to Africans as an equal right of all, regardless of gender, social or economic status.

Our analysis provides evidence in support of BSE as a viable strategy for downstaging BC in Africa. Patients who detect breast masses during BSE are less likely to delay (Devi et al., 2007), perhaps due to selection bias and willingness to take charge of their health, which might also be an element of planned behavior. Performance of BSE/CBE is low in Africa, and previous research found that 10% of inadvertent lump detections were already locally advanced, with an high proportion of those detected early progressing before diagnosis (30% after 30 days delay, and 70% after 90 days delay) (Agodirin et al., 2020). BSE’s effectiveness for downstaging or improving BC survival is controversial (Miller and Baines, 2011; Corbex et al., 2012), especially in places where most patients present early with non-clinically detectable tumors. Nonetheless, increasing performance of BSE and CBE (Miller, 2008; Romanoff et al., 2017) has potential to reduce the prevalence of late-stage disease. Reducing the number of BCs that progress during the interval between detection and diagnosis is also of utmost importance. Strategies for this have been demonstrated in Sudan where trained indigenous volunteers screened rural communities (Abuidris et al., 2013), and in Ghana where screening targeted micro-communities of BC patients (Bonsu and Ncama, 2019).

Reports of prolonged delay of more than three months are prevalent in Africa, ranging up to 70% compared to 16-17% in Europe and the USA (Innos, Padrik, Valvere, et al., 2013). Breast cancer progression is time-dependent (Fujii et al., 2015; Agodirin et al., 2020); however, the significance of 3 months delay as a predictor of advanced-stage diagnosis was not found in this analysis. The association between delay and advanced stage diagnosis has also not been consistent in African literature; some researchers found an association between long intervals and advanced-stage diagnosis (Brinton et al., 2017) while others did not (Galukande et al., 2014). The conflicting finding might be due to methodological issues as information on delay are collected retrospectively. Also, the impact of delay is usually more clearly demonstrated where most patients present early and with small lesions. 

TNBC and HER2 positive BC are non-modifiable factors associated with advanced-stage diagnosis. Prompt diagnosis and access to effective treatment have potential to improve outcomes as the poor survival outcomes in SSA are also a reflection of inadequate and ineffective treatment (McCormack et al., 2020). Accurate pathologic diagnosis, including immunohistochemistry, navigation through the healthcare system, and receipt of of guideline-concordant treatment, including systemic and locoregional therapy, are necessary to improve outcomes in these subgroups.

This is the first meta-analysis to compare the determinants of delayed presentation and advanced stage presentation between early presenters and late presenters using data from Africa. Unfortunately, the small number of studies, the small sample size available for some of the analyzed variables, and inability to assess health systems-related factors limit our results. There was also significant heterogeneity in some of the results. Heterogeneity reduced significantly after the subgroup analysis, meaning some of the differences might be due to regional factors such as cultural, socio-demographic differences, and differences in health care structures. 

Our results showed paradoxical relationships between some determinants of delay and advanced-stage diagnosis. In this case, preference should be given to factors associated with advanced-stage diagnosis as they are more consistent than determinants of delay lengths. Furthermore, the disease stage is a stronger and more reliable prognostic indicator of outcome than delay (McCormack et al., 2020).

In conclusion, providing quality education to raise BC knowledge, and increasing the prevalence of breast health awareness, BSE, and CBE are promising targets for reducing delays and downstaging BC throughout Africa. Future interventions addressing social and demographic barriers should implement innovative approaches to identify the context-specific determinants and deliver tailored messaging.

## Author Contribution Statement

Agodirin, Aremu, Rahman, and Olatoke conceived and designed the study. Agodirin and Aremu collected articles and extracted data. Agodirin analyzed the data. All authors contributed to the interpretation of the data. Agodirin, Akande, Olaogun, and Romanoff contributed to the draft and writeup of the manuscript. All authors contributed to the review and editing of the manuscript, and all authors contributed to the decision to submit the manuscript.

## References

[B1] Abuidris DO, Elsheikh A, Ali M (2013). Breast-cancer screening with trained volunteers in a rural area of Sudan: a pilot study. Lancet Oncol.

[B2] Adly S, Hewedi IH, Mokhtar NM (2010). Clinicopathologic significance of molecular classification of breast cancer: relation to nottingham prognosis index. J Egypt Natl Canc Inst.

[B3] Agarwal S, Ying J, Boucher KM (2017). The association between socio-economic factors and breast cancer-specific survival varies by race. PLoS One.

[B4] Agbo SP, Khalid A, Oboirien M (2015). Clinical presentation, prevalence and management of breast cancer in Sokoto, Nigeria. J Womens Health Care.

[B5] Agodirin O, Olatoke S, Rahman G (2017). Delay between breast cancer detection and arrival at specialist clinic preliminary revelations of multicentered survey in Nigeria. Texila Int Jl Public Health.

[B6] Agodirin O, Olatoke S, Rahman G (2019). Impact of primary care delay on progression of breast cancer in a black African population: A Multicentered Survey. J Cancer Epidemiol.

[B7] Agodirin O, Olatoke S, Rahman G (2020). Presentation intervals and the impact of delay on breast cancer progression in a black African population. BMC Public Health.

[B8] Akanbi O, Oguntola S, Adeoti M (2015). Delay presentation of breast cancer: a study among south western Nigerian women. Int J Curr Res.

[B9] Akinkuolie AA, Etonyeaku AC, Olasehinde O (2016). Breast cancer patients’ presentation for oncological treatment: a single centre study. Pan Afr Med J.

[B10] Ali R, Mathew A, Rajan B (2008). Effects of socio-economic and demographic factors in delayed reporting and late-stage presentation among patients with breast cancer in a major cancer hospital in South India. Asian Pac J Cancer Prev.

[B11] Austoker J, Bankhead C, Forbes LJ (2009). Interventions to promote cancer awareness and early presentation: systematic review. Br J Cancer.

[B12] Ayoade B, Salami B, Agbola A (2015). Beliefs and practices associated with late presentation in patients with breast cancer; an observational study of patients presenting in a tertiary care facility in Southwest Nigeria. J Afr Cancer.

[B13] Benbakhta B, Tazi M, Benjaafar N (2015). Determinants of patient and health system delays for women with breast cancer in Morocco, 2013. Rev Epidemiol Sante Publique.

[B14] Bish A, Ramirez A, Burgess C (2005). Understanding why women delay in seeking help for breast cancer symptoms. J Psychosom Res.

[B15] Bonsu AB, Ncama BP (2019). Integration of breast cancer prevention and early detection into cancer palliative care model. PLoS One.

[B16] Bray F, Ferlay J, Soerjomataram I (2018). Global cancer statistics 2018: GLOBOCAN estimates of incidence and mortality worldwide for 36 cancers in 185 countries. CA Cancer J Clin.

[B17] Brinton L, Figueroa J, Adjei E (2017). Factors contributing to delays in diagnosis of breast cancers in Ghana, West Africa. Breast Cancer Res Treat.

[B18] Burson AM, Soliman AS, Ngoma TA (2010). Clinical and epidemiologic profile of breast cancer in Tanzania. Breast Dis.

[B19] Cacala SR, Gilart J (2017). Factors relating to late presentation of patients with breast cancer in Area 2 KwaZulu-Natal, South Africa. J Glob Oncol.

[B20] Campbell J, Pyer M, Rogers S (2016). Promoting early presentation of breast cancer in women over 70 years old in general practice. J Public Health (Oxf).

[B21] Corbex M, Burton R, Sancho-Garnier H (2012). Breast cancer early detection methods for low and middle income countries, a review of the evidence. Breast J.

[B22] Deeks A, Lombard C, Michelmore J (2009). The effects of gender and age on health related behaviors. BMC Public Health.

[B23] Devi BC, Tang TS, Corbex M (2007). Reducing by half the percentage of late-stage presentation for breast and cervix cancer over 4 years: a pilot study of clinical downstaging in Sarawak, Malaysia. Ann Oncol.

[B24] Elidrissi Errahhali M, Elidrissi Errahhali M, Ouarzane M (2017). First report on molecular breast cancer subtypes and their clinico-pathological characteristics in Eastern Morocco: series of 2260 cases. BMC Womens Health.

[B25] Ermiah E, Abdalla F, Buhmeida A (2012). Diagnosis delay in Libyan female breast cancer. BMC Res Notes.

[B26] Espina C, McKenzie F, dos-Santos-Silva I (2017). Delayed presentation and diagnosis of breast cancer in African women: a systematic review. Ann Epidemiol.

[B27] Fitzpatrick MB, Rendi MH, Kiviat NB (2019). Pathology of Senegalese breast cancers. Pan Afr Med J.

[B28] Fujii T, Yajima R, Morita H (2015). Implication of duration of clinical presentation on tumor progression and short-term recurrence in patients with early breast cancer. Mol Clin Oncol.

[B29] Galukande M, Mirembe F, Wabinga H (2014). Patient delay in accessing breast cancer care in a Sub Saharan African Country: Uganda. Br J Med Med Res.

[B30] Galukande M, Wabinga H, Mirembe F (2015). Breast cancer survival experiences at a tertiary hospital in sub-Saharan Africa: a cohort study. World J Surg Oncol.

[B31] Gebremariam A, Addissie A, Worku A (2019). Time intervals experienced between first symptom recognition and pathologic diagnosis of breast cancer in Addis Ababa, Ethiopia: a cross-sectional study. BMJ Open.

[B32] Grosse Frie K, Kamate B, Traore CB (2018). Factors associated with time to first healthcare visit, diagnosis and treatment, adn their impact on survival among breast cancer patients in Mali. PLoS One.

[B33] Gueye M, Gueye S, Diallo MS (2017). Socio-demographic factors associated with delays in breast cancer. OJOG.

[B34] Hadgu E, Seifu D, Tigneh W (2018). Breast cancer in Ethiopia: evidence for geographic difference in the distribution of molecular subtypes in Africa. BMC Womens Health.

[B35] Ibrahim NA, Oludara MA (2012). Socio-demographic factors and reasons associated with delay in breast cancer presentation: a study in Nigerian women. Breast J.

[B36] Innos K, Padrik P, Valvere V (2013). Identifying women at risk for delayed presentation of breast cancer: a cross-sectional study in Estonia. BMC Public Health.

[B37] Iqbal J, Ginsburg O, Rochon PA (2015). Differences in breast cancer stage at diagnosis and cancer-specific survival by race and ethnicity in the United States. JAMA.

[B38] Islami F, Lortet-Tieulent J, Okello C (2015a). Tumor size and stage of breast cancer in Cote d’Ivoire and Republic of Congo - Results from population-based cancer registries. Breast J.

[B39] Islami F, Lortet-Tieulent J, Okello C (2015b). Tumor size and stage of breast cancer in Côte d’Ivoire and Republic of Congo - Results from population-based cancer registries. Breast J.

[B40] Jedy-Agba E, McCormack V, Adebamowo C (2016a). Stage at diagnosis of breast cancer in sub-Saharan Africa: a systematic review and meta-analysis. Lancet Glob Health.

[B41] Jedy-Agba E, McCormack V, Adebamowo C (2016b). Stage at diagnosis of breast cancer in sub-Saharan Africa: a systematic review and meta-analysis. Lancet Glob Health.

[B42] Jedy-Agba E, McCormack V, Olaomi O (2017). Determinants of stage at diagnosis of breast cancer in Nigerian women: socio-demographic, breast cancer awareness, health care access and clinical factors. Cancer Causes Control.

[B43] Joffe M, Ayeni O, Norris SA (2018). Barriers to early presentation of breast cancer among women in Soweto, South Africa. PLoS One.

[B44] Kazeem A, Jensen L, Stokes CS (2010). School Attendance in Nigeria: Understanding the Impact and Intersection of Gender, Urban-Rural Residence and Socioeconomic Status. Comp Educ Rev.

[B45] Kohler RE, Moses A, Krysiak R (2015). Pathologically confirmed breast cancer in Malawi: a descriptive study: Clinical profile of breast cancer. Malawi Med J.

[B46] Linsell L, Forbes LJ, Kapari M (2009). A randomised controlled trial of an intervention to promote early presentation of breast cancer in older women: effect on breast cancer awareness. Br J Cancer.

[B47] Marcus T, Lunda S, L F (2013). Delayed breast cancer presentation: hospital data should inform proactive primary care. Afr J Prm Health Care Fam Med.

[B48] Mathew A, George PS, Ramadas K (2019). Socio-demographic factors and stage of cancer at diagnosis: A population-based study in South India. J Glob Oncol.

[B49] McCormack V, McKenzie F, Foerster M (2020). Breast cancer survival and survival gap apportionment in sub-Saharan Africa (ABC-DO): a prospective cohort study. Lancet Glob Health.

[B50] McKenzie F, Zietsman A, Galukande M (2018). Drivers of advanced stage at breast cancer diagnosis in the multicountry African breast cancer - disparities in outcomes (ABC-DO) study. Int J Cancer.

[B51] Miguel F, Lopes LV, Ferreira E (2017). Breast cancer in Angola, molecular subtypes: a first glance. Ecancermedicalscience.

[B52] Miller AB (2008). Practical applications for clinical breast examination (CBE) and breast self-examination (BSE) in screening and early detection of breast cancer. Breast Care(basel).

[B53] Miller AB, Baines CJ (2011). The role of clinical breast examination and breast self-examination. Prev Med.

[B54] Mody GN, Nduaguba A, Ntirenganya F (2013). Characteristics and presentation of patients with breast cancer in Rwanda. Am J Surg.

[B55] Montella M, Crispo A, D’Aiuto G (2001). Determinant factors for diagnostic delay in operable breast cancer patients. Eur J Cancer Prev.

[B56] Mousa SM, Seifeldin IA, Hablas A (2011). Patterns of seeking medical care among Egyptian breast cancer patients: relationship to late-stage presentation. Breast J.

[B57] Muchuweti D, Nyandoro G, Muguti E (2017). Factors contributing to delayed breast cancer presentation: A Prospective Study at Parirenyatwa Group of Hospitals, Harare, Zimbabwe 2010-2013. JCTI.

[B58] Nwafor CC, Keshinro SO (2015). Pattern of hormone receptors and human epidermal growth factor receptor 2 status in sub-Saharan breast cancer cases: Private practice experience. Niger J Clin Pract.

[B59] Odongo J, Makumbi T, Kalungi S (2015). Patient delay factors in women presenting with breast cancer in a low income country. BMC Res Notes.

[B60] Olarewaju SO, Oyekunle EO, Bamiro AO (2019). Effect of socio-demographic variables on patient and diagnostic delay of breast cancer at the foremost health care institution in Nigeria. J Glob Oncol.

[B61] Pace LE, Mpunga T, Hategekimana V (2015). Delays in breast cancer presentation and diagnosis at two rural cancer referral centers in Rwanda. Oncologist.

[B62] Rayne S, Schnippel K, Firnhaber C (2017). Fear of treatments surpasses demographic and socio-economic factors in Affecting patients with breast cancer in Urban South Africa. J Glob Oncol.

[B63] Rayne S, Schnippel K, Grover S (2019). Unraveling the South African Breast Cancer Story: The Relationship of Patients, Delay to Diagnosis, and Tumor Biology With Stage at Presentation in an Urban Setting. J Surg Res.

[B64] Romanoff A, Constant TH, Johnson KM (2017). Association of previous clinical breast examination with reduced delays and earlier-stage breast cancer diagnosis among women in Peru. JAMA Oncol.

[B65] Sayed S, Moloo Z, Wasike R (2018). Ethnicity and breast cancer characteristics in Kenya. Breast Cancer Res Treat.

[B66] Sengal AT, Haj-Mukhtar NS, Elhaj AM (2017). Immunohistochemistry defined subtypes of breast cancer in 678 Sudanese and Eritrean women; hospitals based case series. BMC Cancer.

[B67] Seshie B, Adu-Aryee NA, Dedey F (2015). A retrospective analysis of breast cancer subtype based on ER/PR and HER2 status in Ghanaian patients at the Korle Bu Teaching Hospital, Ghana. BMC Clin Pathol.

[B68] Sow M (2017). Figures of the week: Africa, education, and the 2018 World Development Report. Africa in Focus.

[B69] Stapleton JM, Mullan PB, Dey S (2011). Patient-mediated factors predicting early- and late-stage presentation of breast cancer in Egypt. Psychooncology.

[B70] Tazzite A, Jouhadi H, Saiss K (2013). Relationship between family history of breast cancer and clinicopathological features in Moroccan patients. Ethiopian J Health Sci.

[B71] The Africa-American Institute (2015). A report card on the progress, opportunities and challenges confronting the African education sector.

[B72] Ukah C, Chianakwana G, Ebubedike A (2017). The immunohistochemical profile of breast cancer in Indigenous women of Southeast Nigeria. Ann Med Health Sci Res.

[B73] Youngblood VM, Nyirenda R, Nyasosela R (2020). Outcomes and prognostic factors for women with breast cancer in Malawi. Cancer Causes Control.

[B74] Zhang H, Wang G, Zhang J (2019). Patient delay and associated factors among Chinese women with breast cancer: A cross-sectional study. Medicine.

